# Phenotypic and genotypic analysis of *Candida albicans* vaginal isolates reveals that *ECE1* expression underpins pathogenicity

**DOI:** 10.1128/iai.00304-26

**Published:** 2026-06-22

**Authors:** Amirhossein Davari, Junyan Liu, Jabez P. Fortwendel, Arturo Luna-Tapia, David J. Lowes, Jian Miao, Hubertine M. E. Willems, Christian A. DeJarnette, Tracy L. Peters, Nasim Ahmadi, Surabhi Naik, Marc Swidergall, P. David Rogers, Jack D. Sobel, Glen E. Palmer, Brian M. Peters

**Affiliations:** 1 Pharmaceutical Sciences Graduate Program, University of Tennessee Health Science Center 12326, Memphis, Tennessee, USA; 2 Department of Clinical Pharmacy and Translational Science, College of Pharmacy, University of Tennessee Health Science Center 12326, Memphis, Tennessee, USA; 3 College of Light Industry and Food Science, Guangdong Provincial Key Laboratory of Lingnan Specialty Food Science and Technology, Academy of Contemporary Agricultural Engineering Innovations, Zhongkai University of Agriculture and Engineering 47894, Guangzhou, China; 4 Division of Infectious Diseases, Harbor-UCLA Medical Center 21640, Torrance, California, USA; 5 The Lundquist Institute for Biomedical Innovation at Harbor-UCLA Medical Center 117316, Torrance, California, USA; 6 Department of Pharmacy and Pharmaceutical Sciences, St Jude Children's Research Hospital 5417, Memphis, Tennessee, USA; 7 Research Bioinformatics Core, Rush University 2461, Chicago, Illinois, USA; 8 David Geffen School of Medicine at University of California, Los Angeles 722744 , Los Angeles, California, USA; 9 Department of Internal Medicine, Wayne State University School of Medicine 12267, Detroit, Michigan, USA; 10 Department of Microbiology, Immunology, and Biochemistry, University of Tennessee Health Science Center 12326, Memphis, Tennessee, USA; University of California Davis, Davis, California, USA

**Keywords:** candidiasis, vulvovaginal, mucosal, *Candida*, candidalysin, VVC

## Abstract

*Candida albicans* is the leading cause of vulvovaginal candidiasis (VVC), yet the extent to which strain-level variation influences pathogenic mechanisms remains unclear. Candidalysin, a peptide toxin encoded by *ECE1*, is a key driver of epithelial damage and immunopathology, but its role has been largely defined in a limited number of laboratory strains. Here, we analyzed a panel of 27 vaginal clinical isolates obtained from asymptomatic, acute VVC, and recurrent VVC (RVVC) subjects and performed whole-genome sequencing and broad phenotypic profiling, including growth, biofilm formation, stressor and antifungal susceptibility, filamentation, and *ECE1* expression. To directly assess the role of candidalysin, *ece1*Δ/Δ mutant pairs were generated for each isolate and evaluated *in vitro* and in a murine model of VVC. Isolates exhibited substantial, context-dependent phenotypic heterogeneity, and pathogenic traits did not cluster according to clinical classification. Despite this diversity, deletion of *ECE1* universally reduced epithelial damage and inflammatory responses *in vitro* and attenuated immunopathological markers *in vivo*. Notably, the reference strain SC5314 displayed enhanced virulence compared to most clinical isolates, further demonstrating that it represents a hypervirulent outlier. Collectively, these findings demonstrate that the virulence determinant candidalysin operates across a highly heterogeneous population of vaginal clinical isolates, and its expression is positively correlated to pathogenicity. These studies further highlight the importance of delineating virulence attributes beyond the lens of SC5314.

## INTRODUCTION

Despite the overwhelming worldwide incidence of vulvovaginal candidiasis (VVC) caused primarily by the fungal pathogen *Candida albicans*, it currently remains an unmet clinical need (1). It has been estimated that nearly 75% of all women will experience at least one episode of VVC in their lifetime, and 5–8% of all women suffer from recurrent VVC (RVVC), defined as three or more episodes per year (2). Rough calculations based on the age-susceptible population indicate that RVVC alone affects upwards of 100 million women each year globally (3). While not lethal, VVC/RVVC negatively impacts quality of life, resulting in symptoms of vaginal itching, burning, and discharge, accompanied by general malaise (4). Such conditions can translate to psychological impairment, lost income, and significant incurred medical costs for diagnosis and treatment, totaling approximately 15 billion USD globally per annum (5). There are known risk factors for VVC onset, including depletion of vaginal microbial communities by broad-spectrum antibiotics, uncontrolled disease states (e.g., diabetes), and effects of reproductive hormones. However, the pathogenesis of VVC/RVVC largely remains enigmatic, partly due to its multifactorial nature, where inputs from host immunity, vaginal environment, and pathogen each play key roles in the infectious process (4).

Pioneering work by Fidel et al., led to the hypothesis that VVC is an immunopathology where exuberant activation of innate host immunity, including the recruitment of neutrophils to the vagina, results in symptomatic infection (6). Subsequent work has revealed that these neutrophils are ineffective at clearing *C. albicans* from the vaginal environment, partly due to their inhibition by the glycosaminoglycan heparan sulfate abundantly present at the vaginal mucosa (7, 8). However, equally important to the host immune-mediated aspect of disease pathogenesis is the fungus itself, equipped with an array of virulence attributes and metabolic flexibility that impact pathogenic fitness in the vaginal niche (3).

Prior work by our laboratory revealed that genetic mutants of *C. albicans* unable to form elongated and invasive hyphal filaments are incapable of eliciting neutrophil recruitment in a murine model of VVC (9). Later, it was shown that while hyphae were necessary to drive immunopathology, it was the activity of candidalysin, a hypha-specific membrane-disruptive peptide toxin encoded by the *ECE1* gene, that was required to elicit immune activation, tissue damage, and neutrophil recruitment (10). However, these *in vivo* phenotypes have largely been systematically assessed through the lens of the reference isolate SC5314.

While phylogenetic analyses have been extensively used to categorize *C. albicans* into distinct genetic clades, attention has increasingly shifted toward understanding the phenotypic heterogeneity that exists among clinical isolates. For example, recent studies have demonstrated that strains 529L (an oral isolate) and CHN1 (a gut isolate) exhibited significantly attenuated pathogenicity and stable colonization in murine models of oral and gastrointestinal challenge (11–14). Indeed, our laboratory has similarly shown that while 529L colonizes the murine vagina at high levels, it fails to elicit characteristic markers of immunopathology (15). Given these findings, it is intriguing to speculate that isolate-to-isolate phenotypic differences could explain why some women can be stably colonized asymptomatically by *C. albicans*, while others only sporadically develop symptoms or suffer from persistent and recurrent infection. Therefore, the objective of this study was to identify genetic or phenotypic signatures of *C. albicans* isolates that are associated with asymptomatic carriage, acute, or recurrent vaginal infection.

## RESULTS

### Phylogenetic and candidalysin allele analysis in vaginal isolates

**Fig 1:**
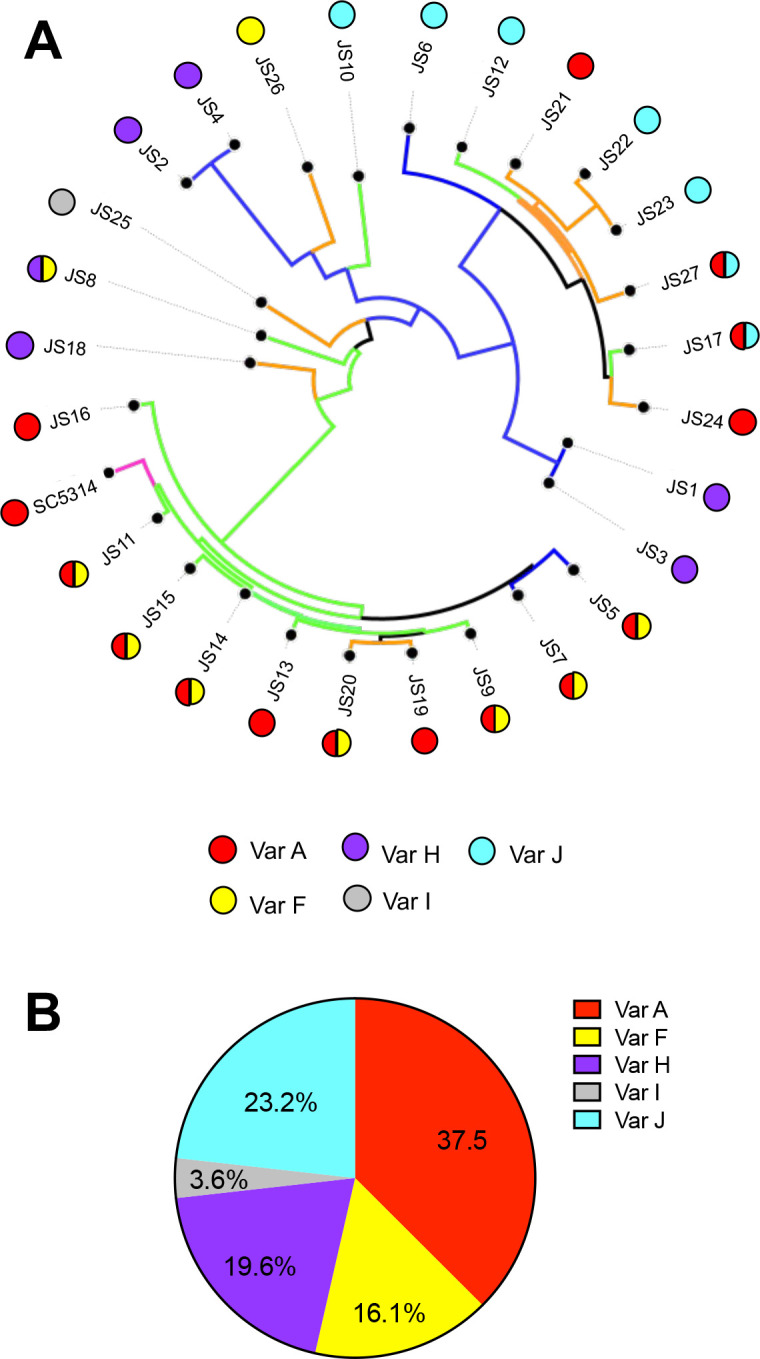
Phylogenetic analysis of *C. albicans* vaginal isolates. (**A**) Single-nucleotide polymorphisms were identified in isolates obtained from asymptomatic (JS1-7, blue), VVC+ (JS8-17, green), and RVVC+ (JS18-27, orange) women, as well as the reference isolate SC5314 (pink). The radial tree depicts genetic relatedness among isolates, with branch lengths representing evolutionary distance. Colored branches indicate clustering of related isolates. Candidalysin alleles were identified by targeted long-read sequencing of *ECE1* amplicons and depicted by colored circles. Dual-colored circles depict heterozygous alleles. (**B**) The frequency of each candidalysin variant observed in this strain collection (*n* = 56 total).

To delineate fungal factors that may be relevant to disease pathogenesis, we employed a panel of 27 vaginal clinical isolates obtained at the Vulvovaginitis Clinic at Wayne State University that were categorized as originating from asymptomatic (*n* = 7), acute VVC+ (*n* = 10), or RVVC+ (*n* = 10) women. Phylogenetic analysis of these clinical isolates and the reference strain SC5314 was carried out using two independent approaches. Analysis based on whole-genome sequences (Fig. 1A) or multi-locus sequence typing (Fig. S1) revealed no distinct phylogenetic patterns that distinguished clinical isolate groupings. Interestingly, targeted haplotype analysis of sequences encoding known candidalysin variants depicted strong grouping between their distribution and genetic relatedness, with variants A (found in SC5314) and J (found in strain 529L) falling on opposite sides of the dendrogram (Fig. 1A) (15, 16). While our sample size was comparatively smaller, candidalysin variant frequency generally matched that reported by Wickramasinghe et al., with variants A, F, H, and J most frequently observed (Fig. 1B and Table S1) (16).

### Growth kinetics and susceptibility to stressors and antifungals

As phylogenetic analysis did not reveal any clear association with infection status, we set out to extensively phenotype this strain collection for a variety of relevant factors that may impact the host-pathogen interaction during VVC. Most strains grew similarly in YPD medium at 30°C, with no apparent differences between isolate groups (Fig. S2). Despite the lag in growth of JS4, JS13, JS15, JS26, and JS27, they reached a comparable OD_600_ to the other strains at the experimental endpoint. Additional phenotypic profiling revealed that most strains were generally insensitive to growth in the presence of the cell wall stressor Congo Red, apart from JS2, JS4, JS13, and JS18 (Fig. S3). Several of the asymptomatic (e.g., JS2, JS4, JS5, and JS6) and acute VVC isolates (JS8, JS10, and JS13) were susceptible to 0.025% or 0.05% SDS, whereas the RVVC isolates were mostly unaffected. Interestingly, approximately 60% of the clinical isolates exhibited increased growth relative to SC5314 in the presence of 5 µg/mL fluconazole. However, this reduced fluconazole sensitivity was evenly distributed across the isolate clinical groupings, demonstrating no clear trend (Fig. S3). Antifungal susceptibility testing revealed that only JS22 and JS23 were considered resistant or dose-dependent susceptible to the first-line azole drug fluconazole when grown at pH 7 (Table S3). These phenotypes were reduced to sensitive when grown at pH 4.5, reflective of the human vaginal environment. However, generally pH did not alter minimum inhibitory concentration (MIC_50_) values beyond a dilution for most isolates.

### Heterogeneity in biofilm formation and hyphal growth

**Fig 2:**
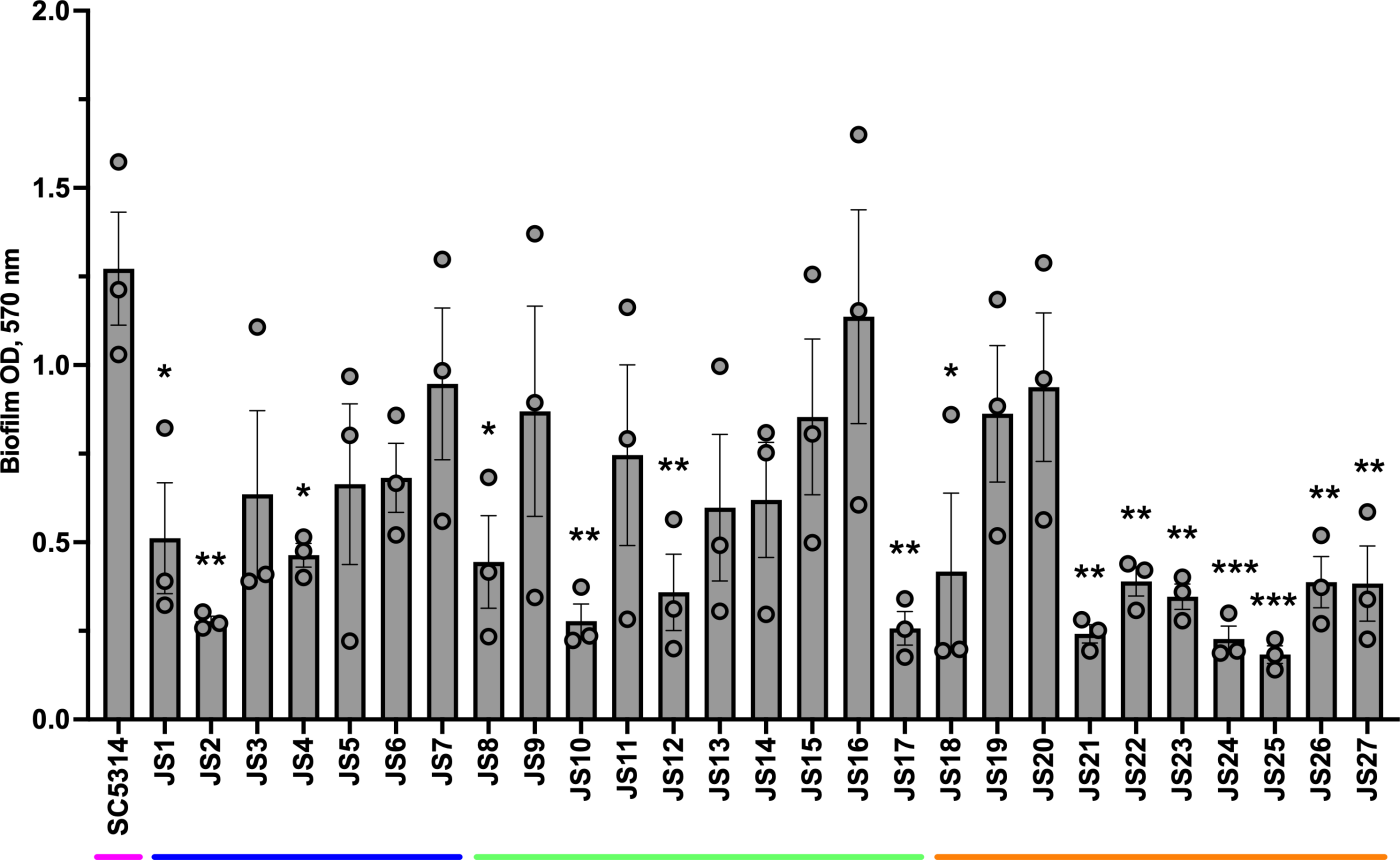
Biofilm formation among *C. albicans* clinical isolates. Biofilm formation by SC5314 (pink) and clinical isolates (asymptomatic: blue; VVC: green; and RVVC: orange) was quantified using a crystal violet assay. Biofilm biomass was measured by absorbance at 570 nm. The data (*n* = 3 independent experiments) are depicted as the mean ± SEM. A one-way analysis of variance with Dunnett’s post-test was used for the statistical analyses comparing each isolate to SC5314. *, *P* < 0.05; **, *P* < 0.01; and ***, *P* < 0.001.

*C. albicans* can form robust biofilms on abiotic and biotic surfaces, including the vaginal mucosa (17). Several studies have implicated that the capacity for isolates to form strong biofilm is an important factor governing colonization and active infection (18–20). Therefore, we utilized a standard biofilm growth model on polystyrene microtiter plates in buffered RPMI-1640 to assess biofilm formation at 24 h (21). SC5314 was among the highest biofilm formers (Fig. 2). Biofilm growth varied considerably among clinical groups, with many of the RVVC isolates unexpectedly forming significantly less biofilm (JS21–27). JS2, JS4, JS10, and JS17 also formed poor biofilm.

**Fig 3:**
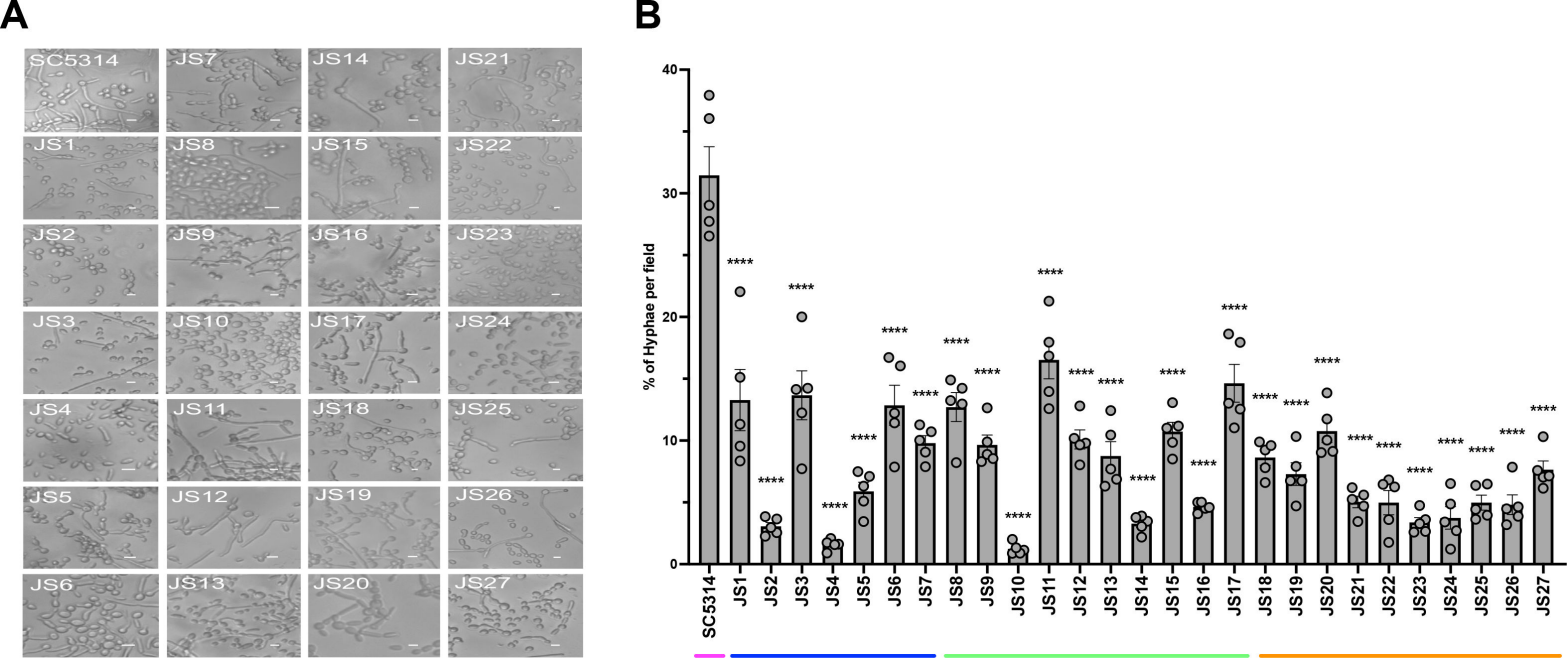
*C. albicans* clinical isolates exhibit variable hyphal growth. (**A**) Representative microscopy images showing hyphal formation of isolates after 24 h static incubation in RPMI-1640 medium. Scale bar, 5 µm. (**B**) Quantification of hyphal growth by SC5314 and clinical isolates (asymptomatic: blue; VVC: green; and RVVC: orange) expressed as the percentage of hyphae per field. The data (*n* = 5 experiments) are depicted as the mean ± SEM. A one-way analysis of variance with Dunnett’s post-test was used for the statistical analyses comparing each isolate to SC5314. ****, *P* < 0.0001.

In addition to an established role in biofilm formation, we have previously shown that the capacity to form hyphae is required for driving the immunopathogenesis of murine VVC. Therefore, we assessed hyphal growth phenotypes across clinical isolates under conditions relevant to commonly used laboratory assays. Buffered RPMI-1640, which is widely used for antifungal susceptibility testing and host–pathogen interaction studies, was selected as the hypha-inducing medium (22, 23). Filamentation was evaluated following standardized inoculum preparation and incubation in RPMI at 37°C for 24 h under static conditions in 96-well plates. This design replicated common macrophage and epithelial challenge models (24, 25). Qualitative (Fig. 3A) and quantitative (Fig. 3B) microscopic analyses revealed that all clinical isolates exhibited reduced hyphal formation compared to the reference strain SC5314. Among these, JS2, JS4, JS10, and JS14 displayed the weakest filamentation (<5% of SC5314), with predominantly yeast morphologies observed. In contrast, several isolates, including JS1, JS3, JS6, JS7, JS8, JS11, JS17, and JS20, exhibited relatively high levels of filamentation (although still lower than SC5314), forming mixed populations of hyphae and yeast. Moreover, these phenotypes were generally consistent with their capacity to form biofilm (Fig. 2). Interestingly, JS8, JS12, and JS17, which demonstrated good hyphal growth, formed poor biofilm. Thus, these data indicate that hyphal growth is insufficient to predict biofilm robustness in clinical isolates.

To determine if filamentation phenotypes were altered during planktonic culture, hyphal growth was also similarly assessed at 4 and 24 h in a variety of media. With the exception of JS2 and JS4, most strains filamented in buffered RPMI-1640 (Fig. S4A and B). Filamentation was also assessed in DMEM, a cell culture medium frequently used for vaginal epithelial cell challenge studies. Interestingly, most strains filamented very poorly after 4 h in this medium, including SC5314 (Fig. S4C). At 24 h, most isolates failed to filament or formed only short germ tubes, except for JS6 and JS8, which more closely resembled filamentation patterns observed in SC5314 (Fig. S4D). Interestingly, all strains robustly filamented, including JS2, JS4, and JS10, and formed large hyphal masses when grown in 10% FBS (Fig. S4E and F). Collectively, these data demonstrated that experimental conditions, including the nutrient microenvironment, drastically influence filamentation phenotypes of clinical isolates and that SC5314 is comparatively hyperfilamentous under most conditions. Moreover, there does not appear to be any consistent trend between isolate groups with respect to filamentation pattern and that the capacity to form hyphae exists along a context-dependent continuum.

### The requirement for *ECE1* to drive epithelial damage and cytokine release is conserved in clinical isolates

**Fig 4:**
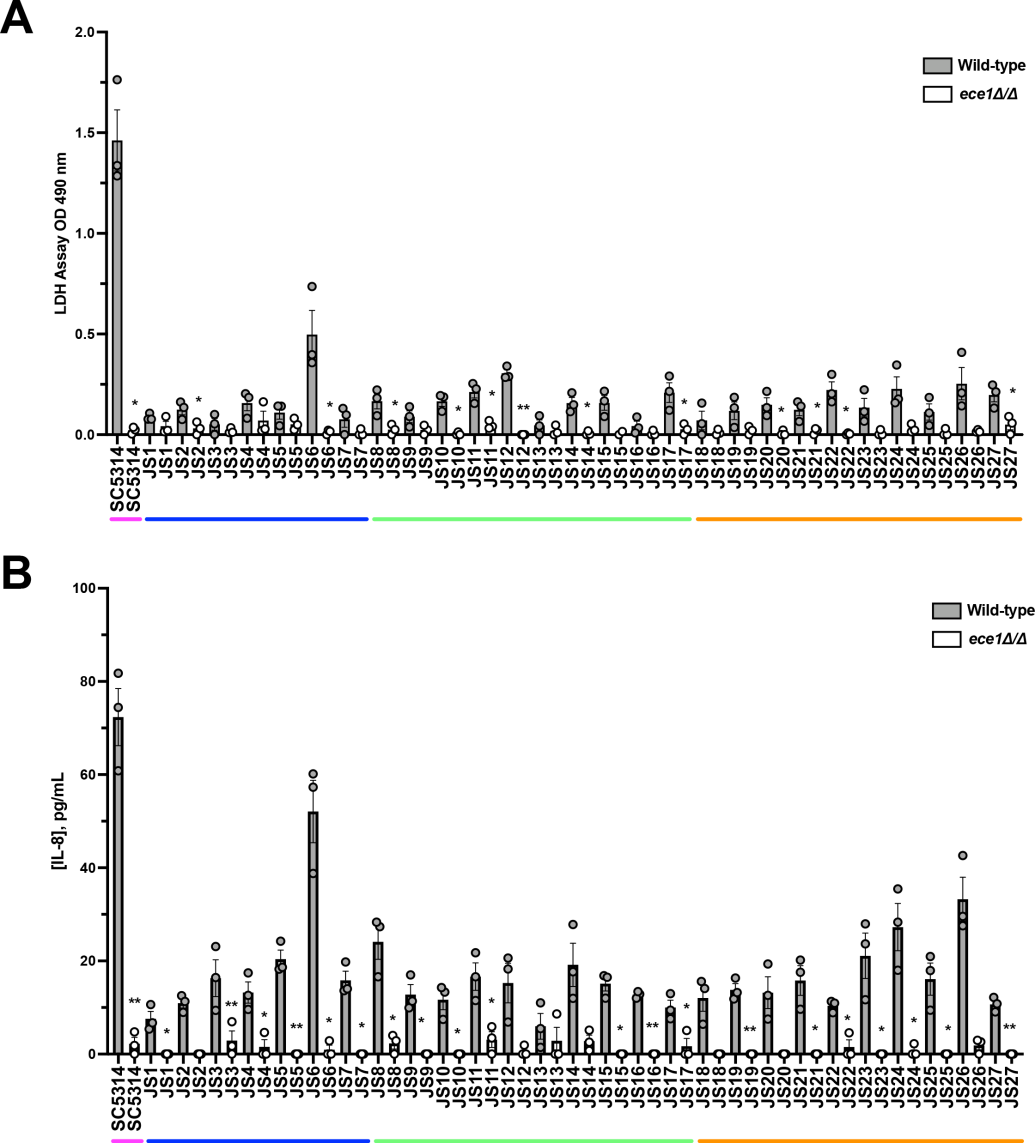
Vaginal epithelial damage and IL-8 responses induced by *C. albicans* clinical isolates. A431 epithelial cells were challenged with *C. albicans* strains (SC5314: pink; asymptomatic: blue; VVC: green; and RVVC: orange) and their corresponding *ece1*Δ/Δ mutants at an MOI of 1:100 (yeast:epithelial cell) for 24 h. (**A**) LDH and (**B**) IL-8 release were measured in culture supernatants by enzymatic assay and ELISA, respectively. The data (*n* = 3 independent experiments) are depicted as the mean ± SEM. Statistical comparisons between each clinical isolate and its corresponding *ece1Δ/Δ* mutant were performed using multiple *t*-tests. *, *P* < 0.05 and **, *P* < 0.01.

We next asked whether pathogenicity and immunostimulatory activity varied among clinical isolates and if these factors were *ECE1*-dependent. Thus, we created *ece1*Δ/Δ deletion mutants in SC5314 and all 27 isolates via CRISPR-Cas9 gene editing (26). A431 vaginal epithelial cells were challenged with each corresponding parental and mutant isolate. All parental strains could drive cytotoxicity as measured by lactate dehydrogenase (LDH) release (Fig. 4A, closed bars) and production of the neutrophil chemotactic cytokine IL-8 (Fig. 4B, closed bars). However, capacity to damage and elicit IL-8 for most isolates was approximately 10–30% of that observed for SC5314. Interestingly, the asymptomatic isolate JS6 was the most pathogenic clinical isolate assessed. Challenge of A431 cells with the corresponding *ece1*Δ/Δ deletion mutants almost completely ablated cytotoxicity (Fig. 4A, open bars) and capacity to drive IL-8 release (Fig. 4B, open bars). Collectively, these data demonstrate that *ECE1* (and likely candidalysin) is a conserved virulence determinant across disparate clinical isolates that is required for driving pathogenicity and immune stimulation of vaginal epithelial cells.

**Fig 5:**
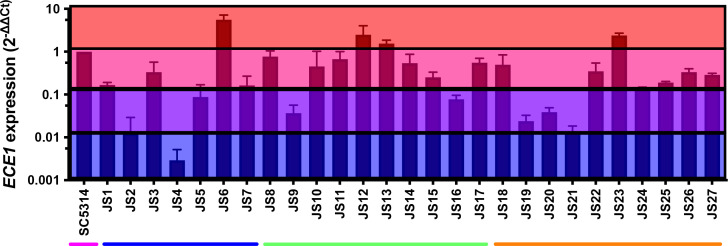
*ECE1* expression among vaginal isolates**.** Expression of *ECE1* in SC5314 (pink) and clinical isolates (asymptomatic: blue; VVC: green; and RVVC: orange) was quantified by RT-qPCR following growth under hypha-inducing conditions. RNA was isolated after 4 h of incubation in buffered RPMI at 37°C, and *ECE1* transcript levels were determined using SYBR Green–based qPCR. Expression levels were normalized to *ACT1* and relative to SC5314 using the 2^−ΔΔCt^ method. The data (*n* = 2 independent experiments) are depicted as the mean + SEM.

We next assessed whether variation in epithelial damage and IL-8 production among clinical isolates was associated with differences in *ECE1* expression. Therefore, we sought to determine *ECE1* expression following 4 h of growth in RPMI, a condition which generally supported hyphal growth in most isolates (Fig. 5). Compared to SC5314, most clinical isolates expressed 5- to 10-fold less *ECE1*, with only JS6, JS12, JS13, and JS23 expressing higher levels. JS2 and JS4, which did not filament well in RPMI, expressed 100- and 500-fold less *ECE1*, respectively. RVVC isolates JS19, JS20, and JS21, and also poorly expresses *ECE1* comparatively. Surprisingly, there was no clear association of expression with clinical condition, as asymptomatic isolates (including JS6) expressed *ECE1* to a substantial level.

### *ECE1* expression levels are uniquely correlated with mucosal damage and IL-8 release

**Fig 6:**
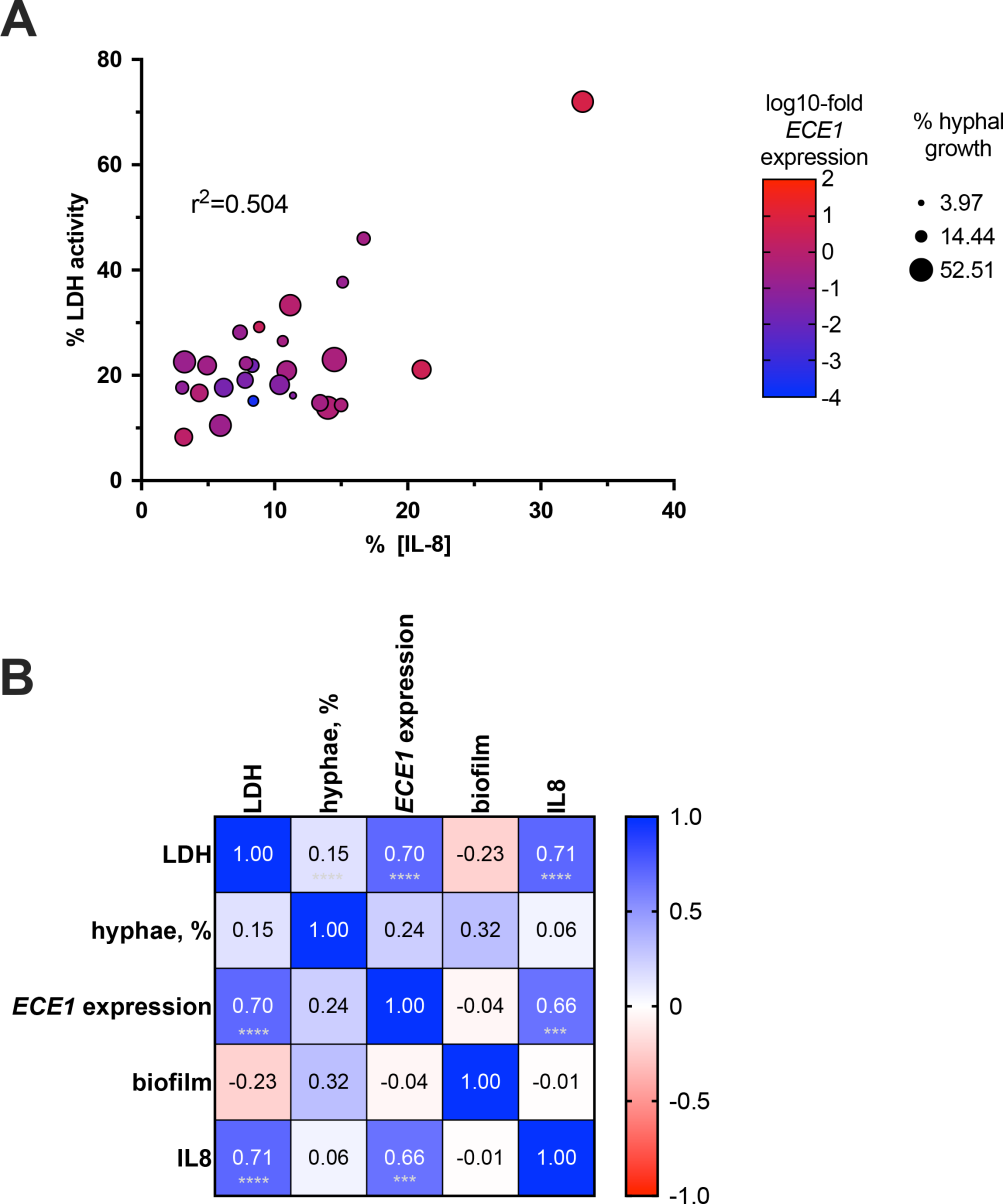
Correlation between epithelial damage, IL-8 release, biofilm formation, hyphal frequency, and *ECE1* expression in clinical *C. albicans* isolates. All data were normalized to SC5314. (**A**) Linear regression analysis comparing IL-8 production and epithelial damage (LDH release) induced by clinical isolates following infection of A431 epithelial cells. Each point represents a single strain. Point size represents the comparative frequency of hyphal growth per field, and color indicates relative log_10_-fold *ECE1* expression. (**B**) Heatmap depicting Pearson R coefficient correlation analysis between LDH release, IL-8 release, biofilm formation, hyphal frequency, and *ECE1* expression. ***, *P* < 0.001 and ****, *P* < 0.0001.

We next integrated these phenotypes to help contextualize our collective observations. All data were normalized to values obtained with the reference isolate SC5314. First, capacity of isolates to cause damage and drive IL-8 release was positively correlated (*r*^2^ = 0.504) (Fig. 6A and B). Second, *ECE1* expression (Fig. 6A, symbol color) was significantly positively correlated with higher damage and cytokine release (Fig. 6B). Generally, strains exhibiting less than 100-fold *ECE1* expression or impaired in filamentation were poor drivers of these responses (Fig. 6A). Interestingly, the frequency of hyphal growth was not significantly associated with cell damage, suggesting that while hyphae are required, the filamentation rate cannot solely predict pathogenicity (Fig. 6A, symbol size). Other strains, like JS13 and JS18 that filamented and expressed *ECE1* comparatively well, were inexplicably unable to cause damage or IL-8 release. In the case of JS13, this could be associated with its relatively slower growth rate denoted above.

### Vaginal immunopathogenesis of clinical isolates is *ECE1*-dependent

**Fig 7:**
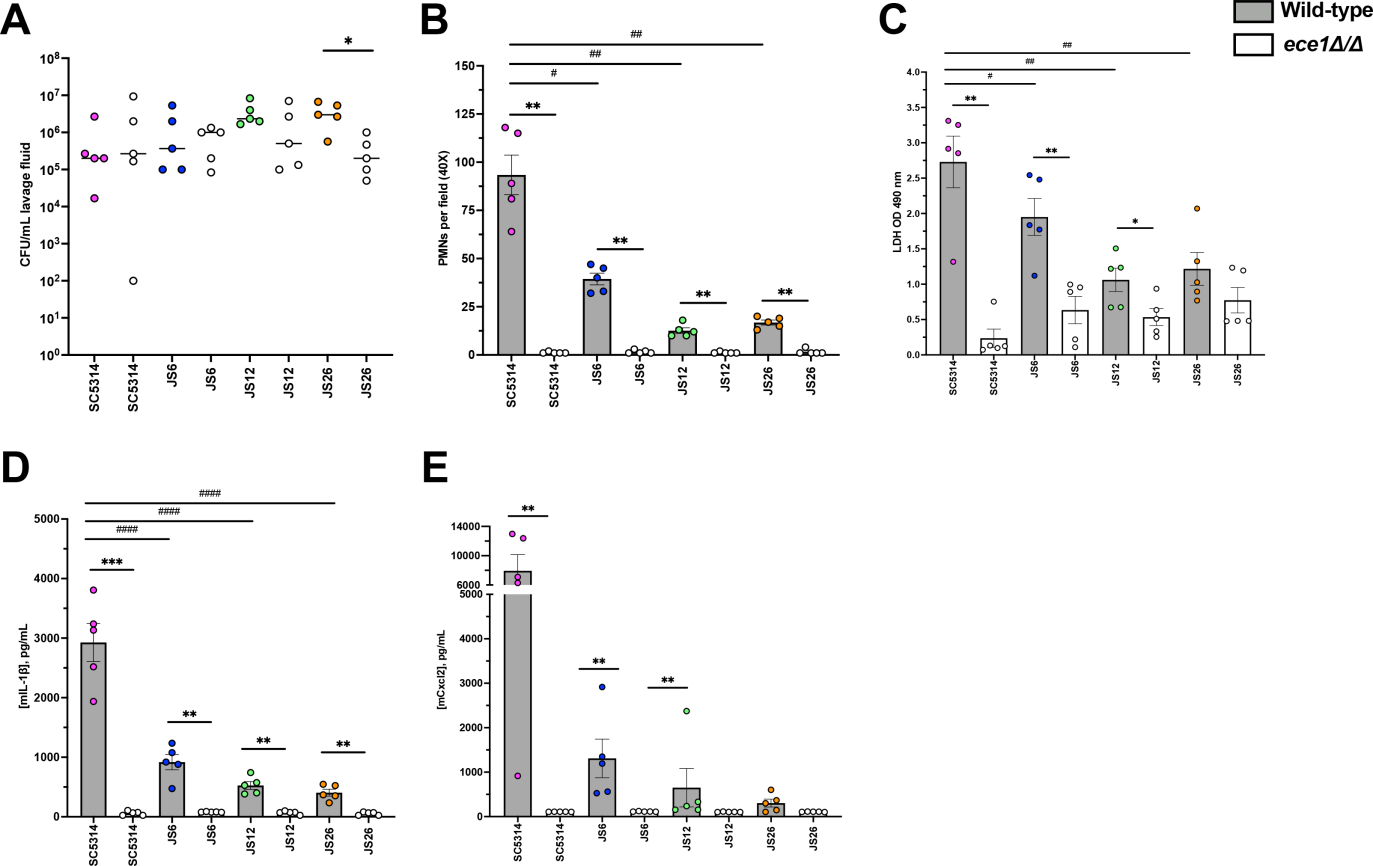
*ECE1* is required for clinical isolates to drive immunopathology in a murine model of VVC. Estrogen-treated mice were intravaginally challenged with representative isolates and their corresponding *ece1*Δ/Δ mutants (SC5314: pink; JS6: blue; JS12: green; and JS26: orange). At day 3 post-inoculation, vaginal lavage fluid (VLF) was assessed for: (**A**) fungal burden by microbiological plating, (**B**) polymorphonuclear leukocytes (PMNs) per field from Papanicolaou-stained vaginal lavage smears, (**C**) epithelial damage as measured by LDH release, and (**D**) IL-1β and (**E**) CXCL2 levels by ELISA. CFU data are depicted as the median and the rest as the mean ± SEM (*n* = 5 mice per group). Statistical comparisons between each clinical isolate and its corresponding *ece1Δ/Δ* mutant were performed using a Mann–Whitney test or multiple *t*-tests depending on normality. Comparison of isolates to SC5314 was performed using a one-way ANOVA with Dunnett’s (parametric) or Kruskal-Wallis (non-parametric) post-tests. *, *P* < 0.05; **, *P* < 0.01; and ***, *P* < 0.001

Finally, we wished to determine how these *in vitro* phenotypes translated to *in vivo* pathogenicity in the murine model of VVC. Therefore, mice were challenged with SC5314, a representative isolate from each clinical group (JS6, JS12, and JS26) that demonstrated damage capacity *in vitro*, and their corresponding *ece1*Δ/Δ deletion mutants. At day 3 post-infection, we observed no major differences in fungal burden between SC5314 and the parental isolates or their *ece1*Δ/Δ mutants, except for JS26 that reached statistical significance (Fig. 7A). Neutrophil (Fig. 7B) and LDH (Fig. 7C) levels found in the vaginal lavage fluid were significantly lower during infection with all clinical isolates as compared to SC5314. Levels of IL-1β (Fig. 7D) and CXCL2 (Fig. 7E), two cytokines previously shown to be elicited in a candidalysin-dependent manner during murine VVC and important for antifungal immunity and neutrophil chemotaxis, were also significantly lower during infection with the clinical isolates as compared to SC5314 (10, 27). Moreover, the magnitude of these collective responses in the clinical isolates generally matched observations during the challenge of A431 cells *in vitro*, where JS6 was the next most pathogenic isolate to SC5314. Importantly, neutrophil chemotaxis, tissue damage, IL-1β, and CXCL2 release were markedly decreased in each *ece1*Δ/Δ deletion mutant compared to its corresponding parental strain (Fig. 7B through E). Interestingly, tissue damage levels were notably higher for the clinical isolate *ece1*Δ/Δ mutants compared to those derived from SC5314. Generally, these parameters held true at day 7 post-infection, except that the clinical isolate *ece1*Δ/Δ deletion mutants began to readily clear from the vaginal cavity, whereas the SC5314 *ece1*Δ/Δ mutant exhibited more stable colonization (Fig. S5).

## DISCUSSION

The characterization of VVC as an immunopathology has provided a framework for improved understanding of disease pathogenesis over the past two decades (28). In the seminal study by Fidel et al., women volunteers were challenged intravaginally with live *C. albicans* and followed for disease symptoms, which correlated with the presence of neutrophils recovered in the vaginal lumen following lavage (6). Strain DB597.94 obtained from an RVVC patient was used as the inoculum. Relatively few of the volunteers (7/19) developed symptoms and only 3/19 presented with cellular infiltrate in vaginal lavage fluid, despite receiving a high inoculum (10^4^–10^7^ blastoconidia). Unfortunately, the virulence profile and phenotypic plasticity of DB597.94 remain uncharacterized. In the results presented here, SC5314 was hypervirulent (robustly forming hyphae and expressing *ECE1*) as compared to nearly every vaginal clinical isolate tested. Thus, it is likely that DB597.94 also exhibits reduced correlates of pathogenicity. It is intriguing to speculate whether challenge with an inherently more virulent strain, such as SC5314, would have driven more consistent symptomatology. Yet our data suggest that robust hyphal growth and strong *ECE1* expression, factors clearly important for driving immunopathology in SC5314, do not correlate well with clinical grouping and that these may be less important for symptomatic clinical presentation (Fig. 6; Table S2). This idea is reflective of recent studies showing strong damaging capacity of vaginal isolates from Italian women, potentially linked with pseudohyphal (as opposed to true hyphal) growth (29, 30). Instead, host genetic susceptibility factors, tolerance to fungal antigen, the local vaginal environment, or additional immunoregulatory mechanisms may play a hierarchical role in governing onset, progression, and immunopathology of human disease (31, 32). In support of this, women without a history of frequent or recurrent vaginal candidiasis were enrolled in the live challenge study out of an abundance of caution for safety reasons (6). Thus, it is plausible that this cohort was already generally less susceptible to symptomatic infection and that the unknown virulence potential of DB597.94 was occasionally sufficient to overcome whatever thresholds may have existed. As our study was pathogen-centric, the equally important population-level variability of host and environmental factors contributing to VVC was not addressed, and their impact remains to be fully delineated.

Through genomic analysis, several studies have suggested that RVVC isolates are longitudinally clonal during subsequent recurrent infections (33–36). However, there is comparatively little information on the diversity of strains that naturally exist during periods of asymptomatic vaginal colonization or the threshold of yeast required to drive a symptomatic response. Thus, it is possible that the most abundant strains routinely isolated are stable colonizers, giving the appearance of clonality, but that subpopulations of more virulent *C. albicans* strains may bloom to initiate symptomatic disease. Additionally, it has been hypothesized that gut colonizing strains may be passively transferred to the vaginal tract via fecal transfer and that transient introduction of potentially virulent strains via this route could provide a nidus for symptomatic infection (37).

Although we did not find any clear phenotype that correlated with clinical grouping, recent work by Sala et al., utilized a human cell culture model to mimic epithelial shedding and showed that isolates associated with symptomatic infection more strongly drove this phenotype, potentially through altered Type I interferon–dependent responses (30). While epithelial shedding was not assessed in our study, the parental clinical isolates that we tested *in vivo* maintained stable colonization through day 7 post-infection, suggesting that even if they are driving epithelial shedding, it is insufficient to reduce murine colonization (Fig. S5). That said, differences between mouse and human epithelial biology or responsiveness to supraphysiologic doses of estrogen may mask this potential impact.

Regarding hyphal growth among isolates, it was quite surprising to observe such heterogeneity with respect to culture medium (Fig. 3; Fig. S4). Most strains tended to filament well in RPMI-1640, apart from JS2, JS4, and JS10. However, all strains, including these, filamented and extensively clumped into hyphal masses when grown in serum. Heightened pathogenicity of vaginal isolates was recently reported during growth in albumin, a component of serum, although these effects were linked to rewired fungal metabolism (24). Thus, caution should be exercised when interpreting hyphal growth phenotypes of clinical isolates, and a multitude of conditions should be evaluated. It was also unexpected to observe very poor filamentation of the clinical isolates in DMEM, a medium routinely used for epithelial-*C. albicans* co-culture (10, 31, 38). While SC5314 robustly filamented, most clinical isolates either formed no hyphae or clusters of stubby filaments. In fact, we adapted our A431 challenge model to utilize RPMI-1640 as described previously because only SC5314 could reproducibly cause damage in DMEM (data not shown) (15, 24). Again, these findings highlight that as more attention is drawn to assessing phenotypic heterogeneity in fungal clinical isolates, experimental systems and approaches will need to be tailored for broad applicability.

On a related note, biofilm phenotypes showed some correlation with hyphal formation under static culture conditions, but this relationship was less apparent when isolates were grown planktonically (Fig. 2; Fig. S4). While strains that were poorly filamentous (e.g., JS2, JS4, and JS10) formed comparatively less biofilm, other strains like JS1 and JS17 formed significantly less biofilm despite displaying good hyphal growth. While the capacity to form hyphae is an established prerequisite for strong biofilm formation, the mechanisms driving this process have been largely studied in the SC5314 background. Thus, it is possible that even in clinical strains that filament well, decreased intensity or duration of expression of adhesins, surface hydrophobicity factors, or matrix production could explain their comparatively poor surface adhesion (39).

Findings between experiments performed in human A431 vaginal epithelial cells were generally recapitulated in the mouse model of VVC, such that the most pathogenic strain (SC5314) drove the highest amount of immunopathologic markers *in vivo*, followed by JS6, JS12, and JS26 in succession (Fig. 4 and 7). Surprisingly, JS6 was categorized as an asymptomatic isolate but was revealed to be the most pathogenic isolate in the collection. Again, this further hints at potential host factors that may overrule pathogenicity traits in relatively low-virulence strains. It is important to note that beyond the designations of asymptomatic, acute VVC, and RVVC, no underlying clinical information exists. For example, it is not guaranteed that “asymptomatic” refers to an isolate that was collected from a woman without a history of infection or if it was obtained during a period of asymptomatic carriage. Similarly, it is unclear when acute VVC and RVVC isolates were obtained—during the symptomatic episode or at any point during a routine visit to the clinic. Thus, more detailed clinical information from a prospective collection effort may help resolve some of the confounding findings observed among clinical groupings.

While candidalysin alleles did not correlate with clinical grouping, it is interesting to note that JS6, JS12, and JS26 all harbored homozygous variant F or J alleles. In the case of JS6, which formed hyphae robustly and highly expressed *ECE1*, the variant J candidalysin allele may partly explain its attenuated pathogenicity relative to SC5314. In prior work from our laboratory, complementation of an SC5314 *ece1*Δ/Δ mutant with an *ECE1* allele harboring a variant J candidalysin attenuated pathogenicity to approximately half of that complemented with an allele containing variant A, reflective of the disparity between SC5314 and JS6 observed here (15). However, additional isolates (e.g., JS16, JS21, and JS24) that harbor strictly variant A candidalysin showed comparatively little damage capacity during A431 challenge, suggesting that the filamentation, *ECE1* expression, and candidalysin allelic variation each contribute to pathogenic potential.

It was also intriguing that *in vivo* LDH release (although lower in all *ece1*Δ/Δ challenges compared to their parental strains) was overall higher in the clinical isolate *ece1*Δ/Δ strains compared to the SC5314 *ece1*Δ/Δ mutant (Fig. 7). We hypothesize that since the morphogenetic program (including hyphal growth and *ECE1* expression) appears to be so aggressively engaged in SC5314, that it has evolved to more strongly rely on the activity of these virulence determinants. Perhaps the comparatively less aggressive commitment to hyphal growth observed in the clinical isolates has resulted in a more balanced reliance of virulence factors, such as secreted proteases and lipases (40–42). Interestingly, clearance of *ece1*Δ/Δ clinical isolates, but not the SC5314 *ece1*Δ/Δ strain, observed by day 7 post-inoculation may also support this hypothesis (Fig. S5). If clinical isolates employ additional virulence determinants more consistently than SC5314, then perhaps this low-level tissue damage is enough to drive removal. If so, it is likely not predicated on the immune effectors quantitated here, as they were universally low during challenge with the *ece1*Δ/Δ deletion strains. An equal possibility is that *ECE1* plays a more prominent role in colonization or adhesion in clinical strains but less so in SC5314. However, comprehensive transcriptional profiling studies would be required to unravel these questions. Regardless, in both the reference and clinical strains, *ECE1* was universally required for full virulence and immune stimulation.

Collectively, we have revealed that genetically diverse vaginal clinical isolates exhibit high phenotypic plasticity with respect to hyphal growth, *ECE1* expression, and pathogenicity *in vitro* and *in vivo*. Moreover, pathogenicity phenotypes cannot be predicted based on clinical assignment or candidalysin allele alone and thus require rigorous testing. Given the strong correlation between findings observed during *in vitro* and *in vivo* challenge, the A431 model using RPMI-1640 medium provides a reliable surrogate to categorize both cytotoxicity and immunostimulatory capacity of clinical isolates. Perhaps most importantly, we reveal that *ECE1* expression level is correlated with pathogenicity and is required for vaginal damage in all strains assessed herein. Finally, aside from offering a phenotypic characterization of a unique strain set, we present 27 new genomes from the human vaginal niche that can be secondarily mined for detailed genotype-by-phenotype analyses.

## MATERIALS AND METHODS

### Fungal strains and isolates

*C. albicans* strain SC5314 (ATCC MYA-2876) was used as the reference isolate for all experiments. De-identified vaginal clinical isolates were obtained from a strain depository housed at the Wayne State University Vaginitis Clinic in Detroit, MI, USA, and labeled as JS1–JS27. Aside from the following descriptors, no other information was provided for these isolates: JS1–JS7 were from asymptomatic carriers, JS8–JS17 were from episodes of acute VVC, and JS18–JS27 were from women with a history of RVVC. All isolates were plated onto CHROMagar *Candida* medium for initial species verification and subsequently underwent whole-genome sequencing. Homozygous *ece1*Δ/Δ deletion mutants were created in each JS isolate as described below. Strain characteristics and comprehensive phenotype data are found in Tables S1 to S3.

### Microorganism growth

All isolates were stored as glycerol stocks at −80°C. An aliquot of stock was streaked on yeast-peptone dextrose (YPD) agar (BD Difco) and incubated at 30°C for 24–48 h to obtain isolated colonies. A single colony was transferred to liquid YPD (BD Difco) medium and incubated overnight at 30°C with shaking at 200 rpm unless otherwise noted.

### Whole-genome sequencing

Genomic DNA was isolated from overnight cultures of YPD grown SC5314 and JS isolates using the YeaStar genomic DNA kit (Zymo Research) according to the manufacturer’s instructions. Library preparation and sequencing reactions were performed at the University of Alabama at Birmingham Heflin Center for Genomic Science. Sequencing libraries were prepared using the Qiagen QIAseq FX DNA kit per the manufacturer’s protocol, and paired-end 300 bp sequencing reads were generated using the Illumina MiSeq platform. Bioinformatics analyses were provided by code4DNA (code4dna.com). Isolate-specific sequence reads were concatenated and aligned to the *C. albicans* reference genome (C_albicans_SC5314_version_A21-s02-m09-r10_chromosomes.fasta) using BWA-MEM v0.717-r188 (43). Alignment files were sorted based on genome coordinates using SAMtools v1.13, and duplicate alignments were marked using the MarkDuplicates command in Picard v2.17.11 (44). Variants were called using FreeBayes v1.1.0 with the diploid population-based model and annotated using SnpEff v4.3r (45). Draft genomes were assembled using SPAdes v3.12 or MaSuRCA v3.3.1 (46, 47).

### Phylogenetic analysis based on whole-genome sequences

Variant call files (VCFs) were merged, indexed, and single-nucleotide polymorphisms extracted using bcftools v1.22.1 (44). The resulting file was converted to FASTA format using vcf2phylip v2.8 (48). IQ-TREE v1.6.12 was then used to construct a phylogenetic tree using 1,000 ultrafast bootstrap replicates to assess node support (49). The resulting tree was visualized using FigTree v1.4.4.

### Phylogenetic analysis based on multi-locus sequencing typing (MLST)

Multi-locus sequence typing (MLST) was performed using genome assemblies in FASTA format. Alleles and sequence types were identified using FastMLST (https://github.com/EnzoAndree/FastMLST) with the *Candida albicans* MLST scheme available in the PubMLST database (50). The scheme includes seven housekeeping genes: *AAT1a*, *ACC1*, *ADP1*, *MPIb*, *SYA1*, *VPS13*, and *ZWF1b* (51). For each isolate, allele numbers at the seven loci were determined and used to generate MLST profiles. Concatenated MLST allele sequences were aligned using MAFFT v7.520, and a phylogenetic tree was constructed using IQ-TREE v1.6.12 with 1,000 ultrafast bootstrap replicates to assess branch support (52). The resulting tree was visualized as a circular phylogeny representing the genetic relationships among the isolates.

### Candidalysin allele identification

Amplicons covering the *ECE1* open reading frame and 5′ and 3′ intergenic sequences were generated using high-fidelity SuperFi II polymerase with primers ECE1FL-F + ECE1FL-R (Table S4), and long-read sequencing was performed via the Oxford Nanopore platform (Plasmidsaurus). Resulting FASTQ files were aligned to the *C. albicans ECE1* reference sequence (*Candida* Genome Database) from strain SC5314 using minimap2 v2.28, then sorted and indexed with SAMtools v1.20 (44, 53). Alignments were inspected with Integrated Genomics Viewer v2.19.7 to visualize heterozygous and homozygous polymorphisms (54). A combination of Longshot v1.0.0 and WhatsHap v2.8 was used to call and phase variants, respectively (55, 56). Consensus candidalysin coding sequences were determined from phased variant call files using bcftools v1.22.1, then extracted and translated using the alternative yeast nuclear codon table with seqkit v2.13.0 (44, 57). Alleles were matched to candidalysin variants identified by Wickramasinghe et al. (16).

### Growth curves

Isolates were grown overnight in YPD, washed three times in sterile water, and diluted to 1 × 10^5^ cells/mL in fresh YPD medium. Suspensions were added to microtiter plates, incubated at 30°C with orbital shaking, and the OD_600_ nm was captured at hourly intervals using a Biotek Synergy spectrophotometer. Values were blank-subtracted using wells containing medium only.

### Stressor assays

Strains were tested for their susceptibility to the cell wall stressor sodium dodecyl sulfate (SDS) and Congo Red (CR), as well as the common antifungal fluconazole (FLU). Strains were grown in YPD, washed three times in sterile water, and resuspended in sterile water at 2.5 × 10^5^ cells/mL. Serial dilutions were performed in a microtiter plate, and a multi-pin tool was used to transfer diluted suspensions to standard YPD plates or those containing 0.025% SDS, 0.05% SDS, 25 µg/mL CR, 100 µg/mL CR, or 5 µg/mL FLU as described previously (58). Plates were incubated for 48 h at 30°C and images captured using a Gel Doc XR Imaging System (Bio-Rad).

### Antifungal susceptibility determination

Antifungal susceptibility testing was performed using the broth microdilution method per the Clinical and Laboratory Standards Institute (CLSI) document M27-A3 with minor modification as described (59). RPMI-1640 medium containing 2% glucose and 165 mM 3-(N-morpholino)propanesulfonic acid (MOPS) was adjusted to either pH 7 or pH 4.5. Fluconazole was diluted in DMSO, adjusted to twice the final starting concentration in the above media, and serially diluted. *C. albicans* strains were grown overnight in YPD at 30°C, streaked for isolation onto Sabouraud Dextrose (SD) agar plates (BD Difco), and incubated overnight at 30°C. Colonies were resuspended in sterile water and turbidity adjusted to a 0.5 McFarland standard. Cell suspensions were further diluted 1:1,000 (~1 × 10^3^ cells/mL) in pH-adjusted RPMI, and 100 µL was transferred to wells of a round-bottom 96-well plate containing an equal volume of diluted antifungal solution. The final concentration of DMSO was 1.0% for all treatments. Drug-free control wells with DMSO alone were included. Plates were statically incubated for 24 and 48 h at 35°C and scanned using a Biotek Cytation 7. Antifungal breakpoints were established using methods reported by Sobel (60).

### Hyphal growth assays

Strains were grown overnight in YPD, washed three times in sterile water, and for static hyphal growth assays, cells were seeded 2.5 × 10^3^ cells/mL into 96-well plates and incubated in Roswell Park Memorial Institute 1640 (RPMI) (Gibco) at 37°C for 24 h under static (non-shaking) conditions. Following incubation, cells were imaged by light microscopy for qualitative and quantitative assessment of filamentation. For hyphal induction in liquid culture, cells were diluted to 1 × 10^6^ cells/mL in RPMI-1640 medium supplemented with 165 mM MOPS, pH 7.0, Dulbecco’s modified Eagle medium (DMEM) (Gibco) without phenol red containing 25 mM HEPES (4-(2-hydroxyethyl)−1-piperazineethanesulfonic acid), or 10% fetal bovine serum (FBS), and 1 mL aliquots were transferred to culture tubes. Cultures were incubated at 37°C with shaking, aliquots removed at 4 or 24 h, and fixed with cold 4% PBS-buffered formalin. Images were captured digitally by standard light microscopy.

### Biofilm growth assay

Strains were grown overnight in YPD, washed three times in sterile water, and diluted to 1 × 10^6^ cells/mL in MOPS-buffered RPMI medium. Aliquots (100 µL) were transferred to 96-well cell-culture treated microtiter plates and incubated at 37°C for 24 h in a humidified chamber. Following incubation, wells were extensively washed with water, and biomass stained using the crystal violet method as described previously (61).

### *ECE1* expression

Strains were grown overnight in YPD medium, washed, and diluted to 1:100 in buffered RPMI. After shaking at 200 rpm for 4 h at 37°C, RNA was extracted using the hot-acid phenol method as described (15). RNA concentrations were calculated by obtaining OD_260/280 nm_ ratio via a NanoDrop, and 200 ng aliquots were treated with RNase-free DNase according to the manufacturer’s instructions (Thermo Fisher). Reverse transcription was carried out using random hexamers and following the Revert Aid (Thermo Fisher) protocol per the manufacturer’s specifications. The 2X Maxima SYBR green kit (Thermo Fisher) was used to amplify 100-bp fragments from 20 ng of generated cDNA using primers ECE1invarQPCR-F + ECE1invarQPCR-R and ACT1invarQPCR-F + ACT1invarQPCR-R (Table S4). Based on whole-genome sequences, primers were designed to be nearly identical matches to *ECE1* and *ACT1* sequences from SC5314 and JS isolates. Reactions were monitored using the Applied Biosystems 7500 thermocycler and software. Expression levels of *ECE1* were normalized to *ACT1* and strain SC5314 using the 2^−ΔΔC*t*^ method (62).

### Human vaginal epithelial infection model

A431 vulvar epidermoid carcinoma cells (ATCC, CRL-1555) were cultured in RPMI medium containing l-glutamine and 10% heat-inactivated FBS (Hyclone) at 37°C and 5% CO_2_ (15). Cells were seeded in 96-well plates at a density of 5 × 10^4^ cells/well. Prior to challenge, confluent A431 epithelial cells were serum-starved overnight, and all experiments were carried out in serum- and phenol red-free RPMI. Epithelial cells were challenged with an MOI of 1:100 (yeast:epithelial cell) for 24 h as described (15, 31). Uninfected controls were also included. Following challenge, plates were gently centrifuged (200 rpm) to settle contents, and cell-free supernatants were recovered. Subsequently, Interleukin 8 (IL-8) and lactate dehydrogenase (LDH) release were measured using a human IL-8 ELISA (Thermo Fisher) and CytoTox 96 Non-Radioactive Cytotoxicity Assay (Promega), respectively (63).

### Deletion of *ece1*Δ/Δ in JS isolates

We utilized a modular and recyclable CRISPR-Cas9-mediated transformation system to create homozygous deletions at *ECE1* loci (26). Repair templates were prepared by PCR amplifying SAT1-flipper and CaHygB-flipper plasmids with primers ECE1CC9KO-F and ECE1CC9KO-R using Phusion high-fidelity polymerase (Thermo Fisher) according to the manufacturer’s instructions (Table S4). PCR products were cleaned by column purification and quantified via NanoDrop. To create *in vitro* assembled Cas9-ribonucleoprotein (RNP) complexes, both clustered regularly interspaced short palindromic repeat (CRISPR) RNAs (crRNAs) and tracrRNA were resuspended in supplied nuclease-free Duplex Buffer (Integrated DNA Technologies [IDT]) to 100 μM prior to use and stored at –20°C. Duplexes for each crRNA were prepared by combining 1 μL of tracrRNA, 1 μL of crECE1up or crECE1down crRNA, and 23 μL of nuclease-free water. Reaction mixtures were incubated at 95°C in a thermocycler for 5 min, chilled for 10 min, and then kept at room temperature. RNP complexes were created by combining 3.6 μL of each crRNA-tracrRNA duplex with 2 μg of Alt-R SpCas9 nuclease V3 and incubating at room temperature for ≥5 min. Briefly, washed and diluted *C. albicans* cells were treated with transformation buffer (10 mM Tris, 1 mM EDTA, and 0.1 M lithium acetate (LiAc)), pH 7.5, for 45 min, followed by incubation with 25 mM dithiothreitol for 30 min. After washing in ice-cold water, cells were washed in ice-cold 1 M sorbitol, which was decanted. Pellets were resuspended in residual liquid and transferred to chilled 0.2 cm gap electroporation cuvettes. RNP complexes and 1 µg of each repair template were mixed with cell suspensions and electroporated at 1.8 kV using a Gene Pulser (Bio-Rad). Cells were immediately diluted in 1 M ice-cold sorbitol, centrifuged at 4,000 rpm for 1 min, supernatants removed, 1 mL YPD added, and transferred to a culture tube at 30°C with shaking for 4–6 h. Cultures were serially diluted, plated onto YPD plates containing 200 µg/mL nourseothricin (NAT) and 600 µg/mL hygromycin B (HygB) (GoldBio), and incubated for 48 h at 30°C. Isolated colonies were grown overnight in YPM (YP medium containing 2% maltose instead of dextrose) to induce cassette excision. The following day, cells were diluted, plated onto YPD plates containing 25 µg/mL NAT and 75 µg/mL HygB, and incubated overnight at 30°C. Small colonies were selected and replica plated on YPD, YPD + 200 µg/ml NAT, and YP + 75 µg/ml HygB or used to start an overnight culture in YPD. Genomic DNA was isolated from overnight cultures, and correct genomic integration of the disruption cassette and excision were confirmed by PCR using primers FLPINTF + ECE1INTR, FLIPINTR + ECE1INTF. *ECE1* deletion was confirmed by PCR using primers ECE1invarQPCR-F + ECE1invarQPCR-R (Table S4). Genomic DNA from the untransformed isolates was used as an internal control.

### Murine model of VVC

All animal experiments were performed in accordance with protocols approved by the Institutional Animal Care and Use Committee (IACUC) at the University of Tennessee Health Science Center in Laboratory Animal Care Unit facilities. The VVC murine model was performed as described previously, with slight modification (9). In short, groups of 6- to 8-week-old female C57BL/6 mice (*n*  =  5) were purchased from Charles River Laboratories and were housed in isolator cages that were mounted on ventilated racks. Mice were subcutaneously administered 0.1 mg β-estradiol 17-valerate (estrogen, E2) (Sigma Aldrich) that was dissolved in sesame oil (Sigma-Aldrich) 3 days prior to vaginal lavage or challenge with *C. albicans*. Thereafter, E2 was administered weekly. Stationary-phase cultures of *C. albicans* strains were washed, counted, and then adjusted to 5 × 10^8^ cells/mL in sterile endotoxin-free PBS. The mice were intravaginally inoculated with 10 μL of cell suspension, which generated an inoculum size of 5 × 10^6^ blastoconidia.

### Assessment of vaginal fungal burden and immunopathology

The recovered vaginal lavage fluids (VLFs) were spiked with 100× EDTA-free protease inhibitors (cOmplete; Roche). Fresh VLF (10 μL) was smeared onto Tissue Path Superfrost Plus Gold slides, allowed to air dry, fixed with CytoPrep spray fixative (Fisher Scientific), and stored at room temperature. The VLFs were then centrifuged, and the supernatants were transferred to new tubes and stored at −80°C. Tubes containing pellets were weighed before and after the removal of the supernatant to calculate the amount of PBS with which to resuspend the pellet for microbiological plating. For the assessment of the fungal burden, lavage fluids were serially diluted, plated on YPD agar plates containing 50 μg/mL chloramphenicol, and subsequently incubated at 30°C for 48 h. The resulting colonies were enumerated and reported as the median. Slides containing fixed lavage fluids were stained using the Papanicolaou technique to enumerate the polymorphonuclear leukocytes (PMNs), as identified by their morphologies, staining appearances, and characteristic trilobed nuclei. For each smear, the PMNs were manually counted in five nonadjacent fields via standard light microscopy, using a 40× objective. Lavage fluids were diluted and assessed for mucosal damage by LDH release using the CytoTox 96 Non-Radioactive Cytotoxicity Assay (Promega). Levels of IL-1β and CXCL2 (murine homolog of human IL-8) were also assessed in vaginal lavage fluid by ELISA (Thermo Fisher) (64).

### Correlation and heatmap analyses

Data were correlated using the Pearson correlation coefficient or linear regression analyses. These analyses and their corresponding heatmaps or *r*^2^ values were calculated with GraphPad Prism v10.1.1.

### Image construction

All graphs were constructed using GraphPad Prism v10.1.1. Composite images were constructed using Microsoft PowerPoint v16.81 or GraphPad Prism v10.1.1. Any adjustments to brightness or contrast were made evenly across the entire image. Figures were rendered for publication using Adobe Photoshop v25.2.0.

### Statistical analyses

Imaging, expression analysis, and antifungal susceptibility testing were conducted in biological duplicate unless otherwise noted. Images are representative of at least five independent fields of view. All other experiments were conducted in at least biological triplicate. Data were assessed for normality using the Shapiro-Wilk test. Normally distributed data were analyzed using the one-way ANOVA and Dunnett’s post-test or multiple *t*-tests. Non-normally distributed data were assessed using the one-way ANOVA and Kruskal-Wallis post-test. All statistical analyses were conducted using GraphPad Prism v10.1.1.

## Data Availability

Whole-genome and targeted Nanopore sequencing files for SC5314 or JS isolates have been deposited in the NCBI Sequence Read Archive under BioProject accession number PRJNA1440429.

## SUPPLEMENTAL MATERIAL

Fig. S1 iai.00304-26-s0001.tif Phylogenetic analysis of clinical *C. albicans* isolates.

Fig. S2 iai.00304-26-s0002.tif Growth kinetics of clinical *C. albicans* isolates.

Fig. S3 iai.00304-26-s0003.tif Stressor susceptibility of *C. albicans* isolates.

Fig. S4 iai.00304-26-s0004.tif Hyphal growth of clinical *C. albicans* isolates during planktonic growth in various media.

Fig. S5 iai.00304-26-s0005.tif *ECE1* is required for clinical isolates to drive immunopathology in a murine model of VVC.

Supplemental material iai.00304-26-s0006.docx Legends for all Supplemental tables and figures.

Table S1 iai.00304-26-s0007.xlsx Candidalysin allele sequences and frequency.

Table S2 iai.00304-26-s0008.xlsx Summary of phenotypic and virulence-associated traits of clinical *C. albicans* isolates.

Table S3 iai.00304-26-s0009.xlsx Antifungal susceptibility testing of vaginal isolates.

Table S4 iai.00304-26-s0010.xlsx Oligonucleotides used in this study.

ASM does not own the copyrights to Supplemental Material that may be linked to, or accessed through, an article. The authors have granted ASM a non-exclusive, world-wide license to publish the Supplemental Material files. Please contact the corresponding author directly for reuse.
