# Supplementary figures and images for: Phenotypic and genotypic analysis of Candida albicans vaginal isolates reveals that ECE1 expression underpins pathogenicity
Source: Infect Immun. 2026 Jun 22;94(7):e00304-26. doi: 10.1128/iai.00304-26 (PMC13367051; doi:10.1128/iai.00304-26)

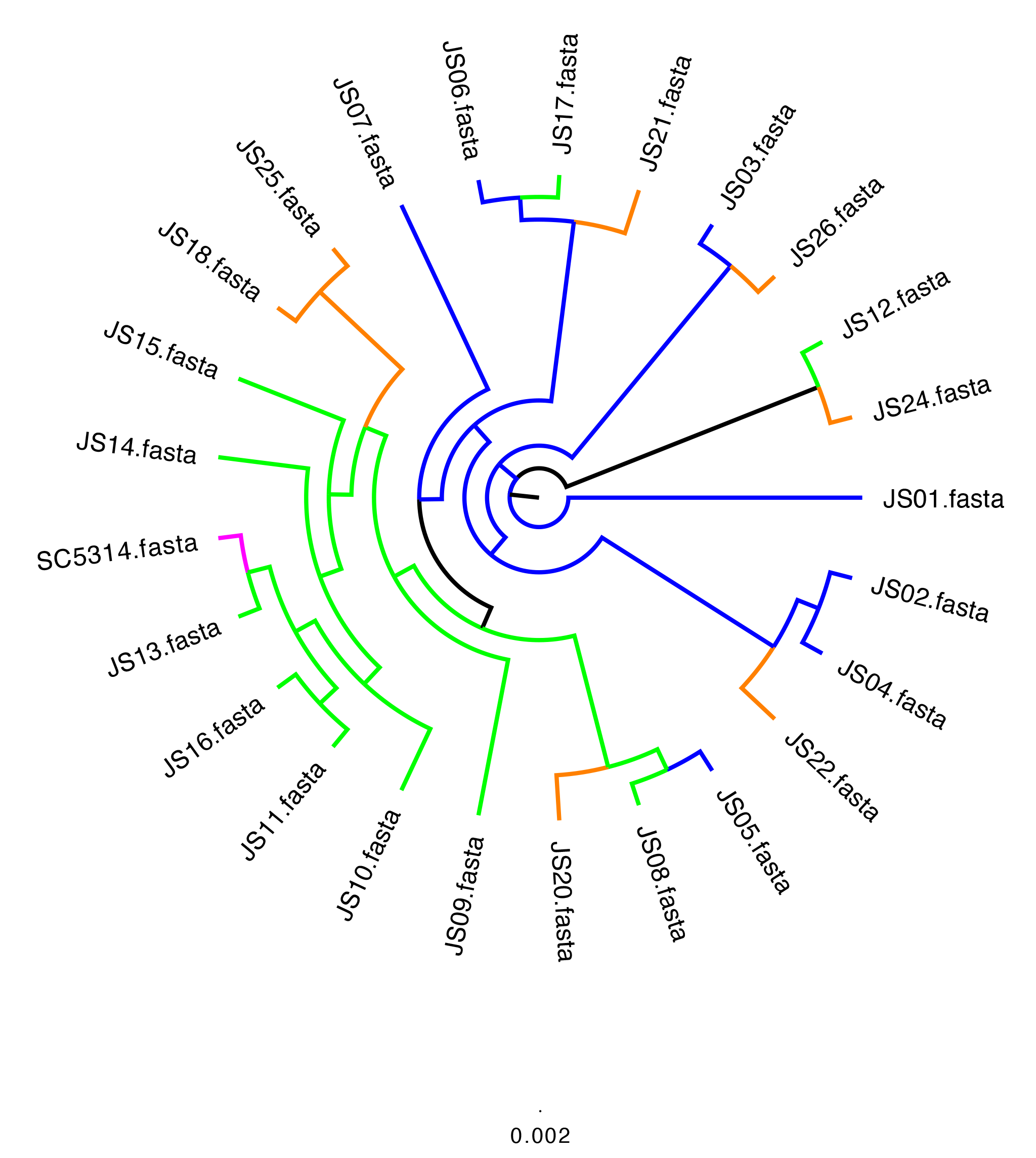

Supplement: Fig. S1 — Phylogenetic analysis of clinical C. albicans isolates. [file iai.00304-26-s0001.tif]

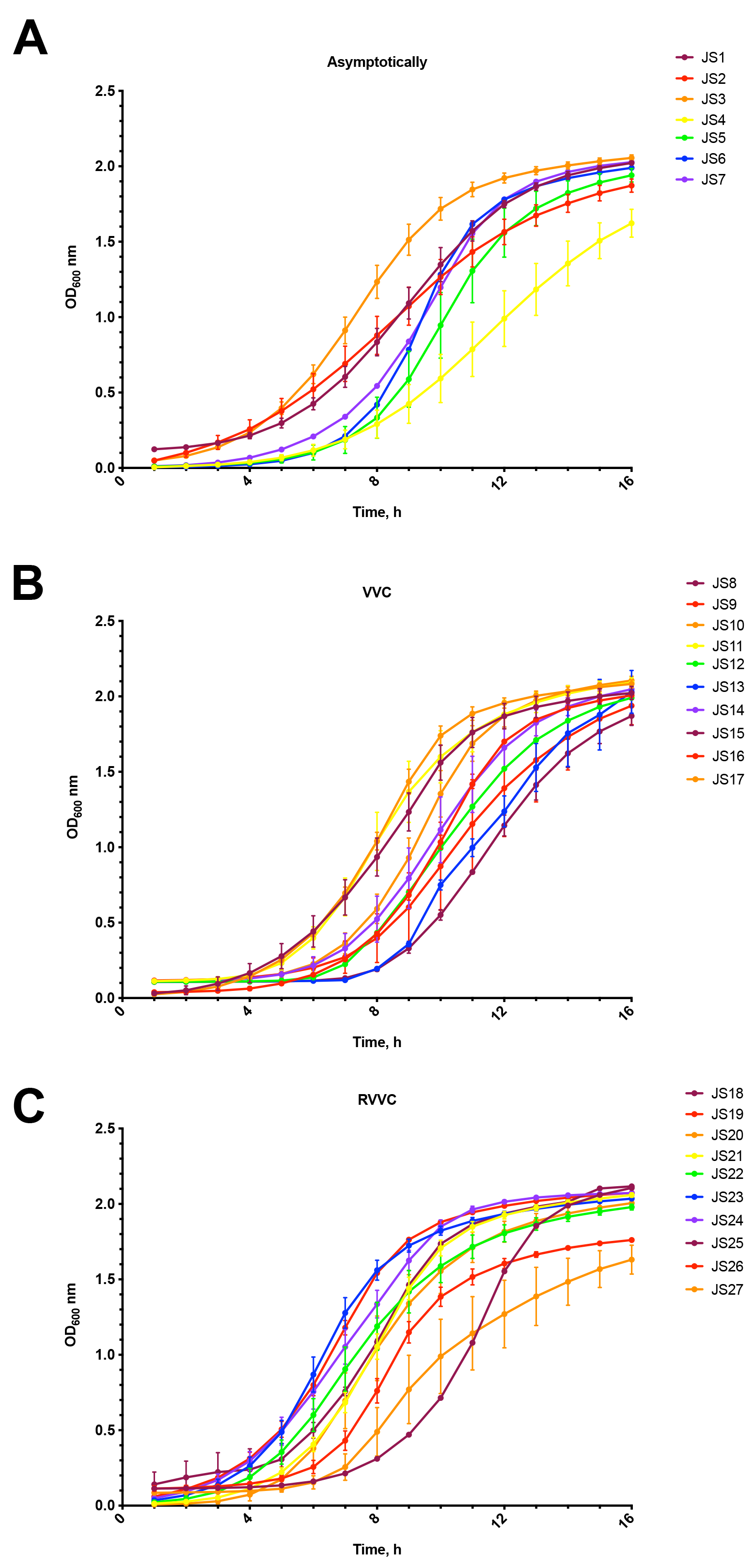

Supplement: Fig. S2 — Growth kinetics of clinical C. albicans isolates. [file iai.00304-26-s0002.tif]

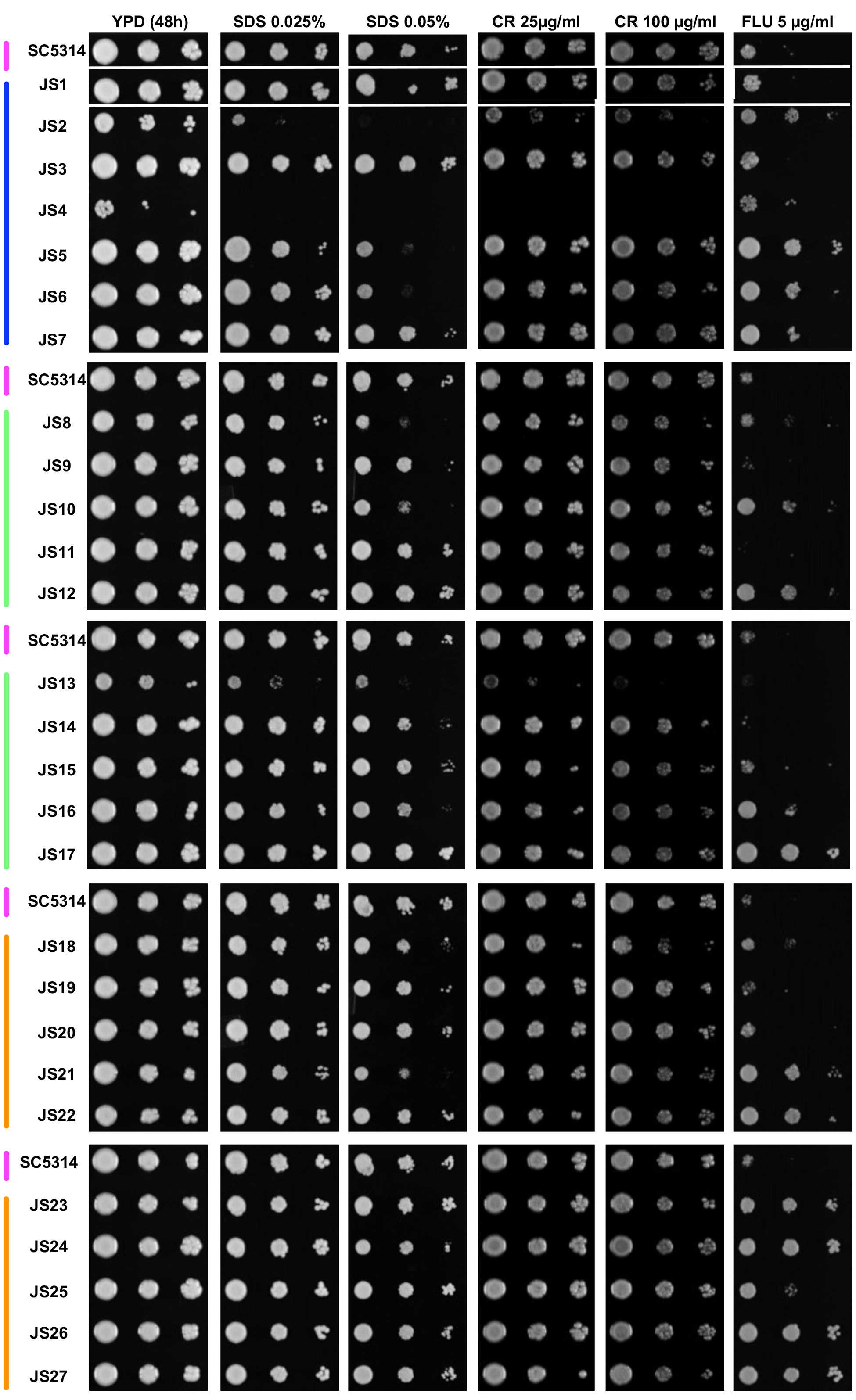

Supplement: Fig. S3 — Stressor susceptibility of C. albicans isolates. [file iai.00304-26-s0003.tif]

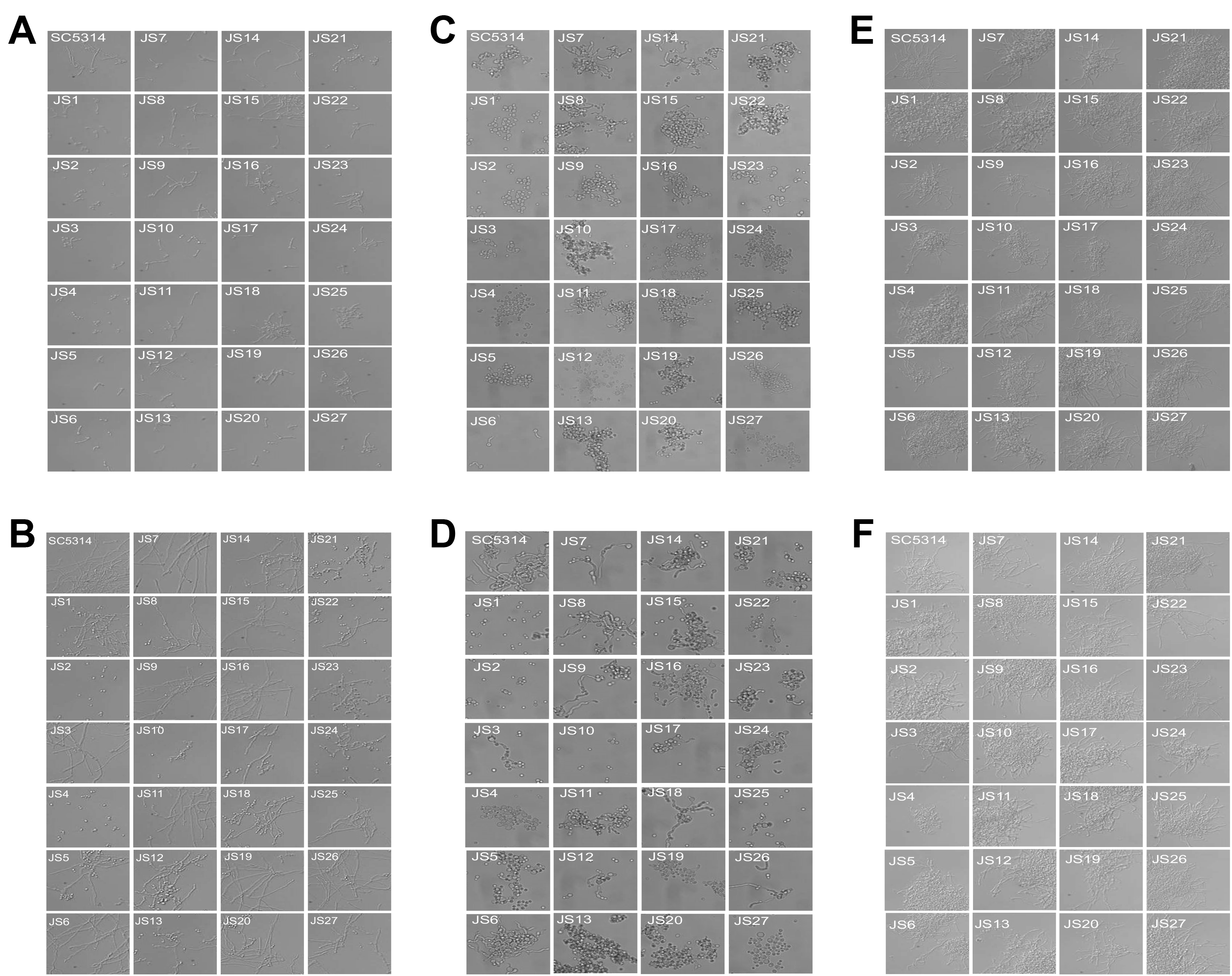

Supplement: Fig. S4 — Hyphal growth of clinical C. albicans isolates during planktonic growth in various media. [file iai.00304-26-s0004.tif]

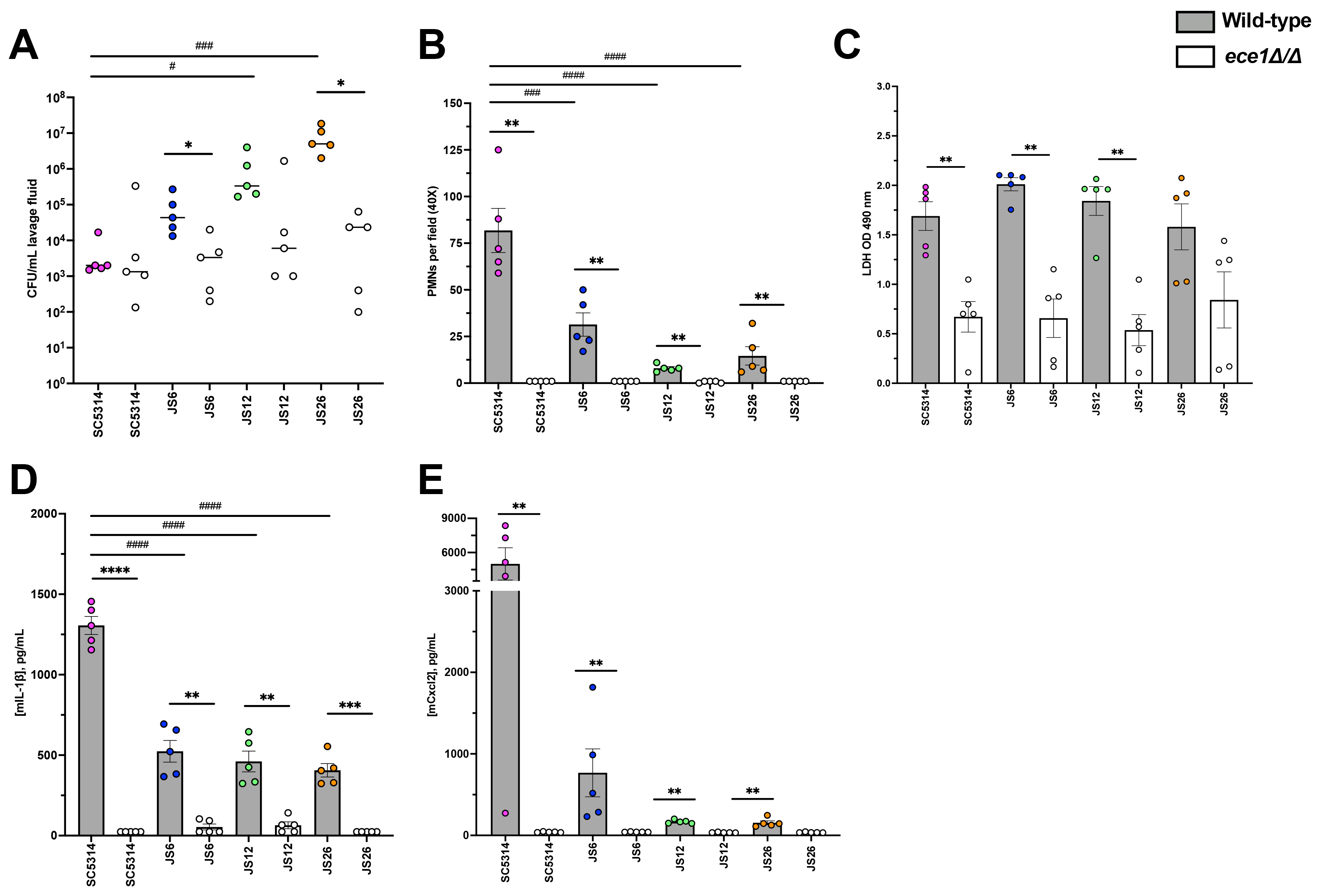

Supplement: Fig. S5 — ECE1 is required for clinical isolates to drive immunopathology in a murine model of VVC. [file iai.00304-26-s0005.tif]
