# Supplementary material for: Phenotypic and genotypic analysis of Candida albicans vaginal isolates reveals that ECE1 expression underpins pathogenicity
Source: Infect Immun. 2026 Jun 22;94(7):e00304-26. doi: 10.1128/iai.00304-26 (PMC13367051; doi:10.1128/iai.00304-26)
Supplement: Supplemental material — Legends for all Supplemental tables and figures. [file iai.00304-26-s0006.docx]

**Supplemental Table 1. Candidalysin allele sequences and frequency.** Candidalysin alleles from SC5314 and clinical isolates were determined by targeted long read sequencing and matched to previously identified variants. Strains in bold have heterozygous candidalysin alleles. The frequency of each allele and percentage represented were also calculated.

**Supplemental Table 2. Summary of phenotypic and virulence-associated traits of clinical *C. albicans* isolates.** Clinical isolates were categorized as from asymptomatic, acute VVC+, or RVVC+ women. Quantitative measurements include static hyphal growth in RPMI-1640 conditions, *ECE1* expression, biofilm formation, epithelial cell damage, and IL-8 production. Qualitative phenotypes include sensitivity to sodium dodecyl sulfate (0.025%), Congo Red (100 µg/mL) and fluconazole (5 µg/mL), as well as filamentation during planktonic growth under different media and time conditions (RPMI-1640, DMEM, and FBS at 4 h and 24 h). “y” indicates presence of the phenotype or sensitivity, and “n” indicates absence.

**Supplemental Table 3. Antifungal susceptibility testing of vaginal isolates.** Susceptibility to fluconazole (at pH 7 and pH 4.5) was determined. Clinical breakpoints are defined. S = susceptible (≤ 2 µg/mL), SDD = susceptible dose-dependent (4 µg/mL), R = resistant (≥ 8 µg/mL).

**Supplemental Table 4. Oligonucleotides used in this study.** All primers and crRNAs are denoted in the 5ʹ🡪3ʹ orientation. Underlined sequences indicate regions that anneal to *SAT1-* and *CaHygB*-flipper plasmids.

**Supplemental Figure 1. Phylogenetic analysis of clinical *C. albicans* isolates.** Multilocus sequence typing was performed on isolates obtained from asymptomatic (JS1-7, blue), VVC+ (JS8-17, green), and RVVC+ (JS18-27, orange) women, as well as the reference isolate SC5314 (pink). The radial tree depicts genetic relatedness among isolates, with branch lengths representing evolutionary distance. Colored branches indicate clustering of related isolates.

**Supplemental Figure 2. Growth kinetics of clinical *C. albicans* isolates.** Clinical isolates were grown in YPD medium and optical density (OD600 nm) was measured over time. Growth curves depict **(A)** asymptomatic isolates (JS1–JS7), **(B)** VVC isolates (JS8–JS17), and **(C)** RVVC isolates (JS18–JS27). Colors were randomly assigned to each strain to improve graph readability. Data (n=3 independent experiments) are presented as mean ± SEM.

**Supplemental Figure 3. Stressor susceptibility of *C. albicans* isolates.**Isolates (SC5314: pink; asymptomatic: blue; VVC: green; RVVC: orange) were assessed for growth under cell wall and antifungal stress conditions using spot dilution assays. Serially diluted cultures were plated on YPD alone or YPD supplemented with SDS (0.025% and 0.05%), Congo Red (CR; 25 and 100 µg/mL), or fluconazole (FLU; 5 µg/mL). Plates were incubated for 48 h and imaged. Representative images (n=2 independent experiments) shown.

**Supplemental Figure 4. Hyphal growth of clinical *C. albicans* isolates during planktonic growth in various media.** Filamentation was assessed for SC5314 and clinical isolates (JS1–JS27) under planktonic growth conditions in multiple media in shaking culture. Representative images (n=2 independent experiments) were captured at 4 h and 24 h in buffered (**A,B**) RPMI-1640, (**C,D**) DMEM, and (**E,F**) 10% FBS. Images were acquired by light microscopy.

**Supplemental Figure 5. *ECE1* is required for clinical isolates to drive immunopathology in a murine model of VVC.** Estrogen-treated mice were intravaginally challenged with representative isolates and their corresponding *ece1*Δ/Δ mutants (SC5314: pink; JS6: blue; JS12: green; JS26: orange). At d 7 post-inoculation, vaginal lavage fluid (VLF) was assessed for: **(A)** fungal burden by microbiological plating, **(B**) polymorphonuclear leukocytes (PMNs) per field from Papanicolaou-stained vaginal lavage smears, **(C)** epithelial damage as measured by LDH release, **(D)** IL-1β and **(E)** CXCL2 levels by ELISA. CFU data are depicted as the median and the rest as the mean ± SEM (n = 5 mice per group). Statistical comparisons between each clinical isolate and its corresponding *ece1Δ/Δ* mutant were performed using a Mann–Whitney test or multiple t-tests when data passed the normality test. Comparison of isolates to SC5314 were performed using a one-way ANOVA with Dunnet’s (parametric) or and Kruskal-Wallis (non-parametric) post-tests. *, *P* < 0.05; **, *P* < 0.01; ***, *P* < 0.001.
